# Human serum albumin nanoparticles for efficient delivery of Cu, Zn superoxide dismutase gene

**Published:** 2007-05-23

**Authors:** Yun Mo, Micheal E. Barnett, Dolores Takemoto, Harriet Davidson, Uday B. Kompella

**Affiliations:** 1 Department of Pharmaceutical Sciences and Department of Ophthalmology and Visual sciences, University of Nebraska Medical Center, Omaha, NE; 2 Department of Biochemistry, Kansas State University, Manhattan, KS; 3 Department of Clinical Sciences, Kansas State University, Manhattan, KS

## Abstract

**Purpose:**

To assess the potential of human serum albumin nanoparticles (HSA NP) as a nonviral vector for ocular delivery of Cu, Zn superoxide dismutase (SOD1) gene.

**Methods:**

Cu, Zn superoxide dismutase (*SOD1*) gene-encapsulated nanoparticles (NP) were developed using human serum albumin (HSA), an endogenous protein, by a desolvation-crosslinking method. The pSOD-loaded HSA NP was evaluated for in vitro release characteristics, stability against DNase I and vitreous humor degradation, cytotoxicity, cellular uptake mechanisms, in vitro transfection efficiency, and in vivo gene expression. In vitro studies employed cultured human retinal pigment epithelial (ARPE-19) cells and in vivo studies employed a mouse model. For cell uptake analysis, fluorescein isothiocyanate (FITC)-labeled human serum albumin (HSA) was used.

**Results:**

Plasmid containing *SOD1* gene was encapsulated in HSA by a desolvation-crosslinking method. Gene-loaded HSA NP has a mean size of 120 nm, zeta potential of -44 mV, and plasmid encapsulation efficiency of 84%. At high crosslinking degree, HSA NP sustained the in vitro release of plasmid over 6 days, and stabilized plasmid DNA against DNase I and vitreous humor degradation. No cytotoxicity was observed in ARPE 19 cells treated with blank HSA NP at concentrations up to 5 mg/ml for 96 h. Cellular uptake of HSA NP was via receptor-mediated endocytosis that involves primarily caveolae-pathways. Confocal analysis indicated rapid endo/lysosomal escape of HSA NP. Further, confocal studies indicated that HSA readily enters the cell nucleus. In vitro, pSOD-HSA NP resulted in more than 80% transfection efficiency in ARPE-19 cells, which was 5 fold higher than Lipofectamine. HSA NP-transfected cells exhibited enhanced SOD1 activity that was 5 fold higher than untreated cells, indicating the overexpression of the functional gene. Intravitreal injection of HSA NP to the mouse eye at a dose of 130 ng of plasmid produced detectable level of fusion protein expression at 48 h, compared to non-detectable expression in control animals.

**Conclusions:**

The HSA NP developed in this study offers a very promising approach for nonviral gene delivery to the retina.

## Introduction

Oxidative stress, the cellular damage caused by reactive oxygen species (ROS), is implicated in a series of ocular diseases such as cataract [1], glaucoma [2], uveitis [3], retrolental fibroplasias [4], diabetic retinopathy [5], and age-related macular degeneration (AMD) [6]. The retina is most susceptible to oxidative damage due to its higher oxygen consumption than any other tissue, exposure to cumulative irradiation, and abundance of photosensitizers and polyunsaturated fatty acids that can initiate cytotoxic chain-reactions [7]. The reactive oxygen species generated in the retina are normally detoxified by antioxidants including superoxide dismutase (SOD), catalase, and glutathione peroxidase [7]. Superoxide dismutase 1 (SOD1) or Cu, Zn SOD, present in the cell cytosol is the most abundant SOD in the eye [8]. SOD1 reduces oxidative stress in the retina by converting free oxygen radicals into hydrogen peroxide, which is further broken down into water by enzymes such as glutathione peroxidase or catalase. SOD1-deficient mice exhibit key symptoms of human AMD, such as drusen, choroidal neovascularization, and retinal pigment epithelium dysfunction, suggesting a critical role for SOD1 in protecting the retina from age-related degeneration and the potential of *SOD1* gene-mediated AMD therapy [9]. The objective of the study was to develop a highly efficient, nontoxic, nonviral vector for the retinal delivery of *SOD1* gene.

Nonviral vectors are considered advantageous for gene therapy due to their ability to minimize the host immune response, which is a major concern for viral vectors. A number of nonviral gene delivery systems, particularly nanoparticle systems, have been developed so far using polymers (e.g., poly (D,L-lactic-co-glycolic acid; PLGA) [10], poly(L-lysine) [11], and chitosan [12]), lipids (e.g., ethyldimyristoylphosphatidylcholine (EDMPC) [13], 1,2-distearyloxy-N,N-dimethyl-3-aminopropane (DSDMA) [14], and 1,2-dioleoyl-sn-glycero-3-trimethylammoniumpropane (DOTAP) [15]), and dendrimers (e.g., polypropylenimine (PPI) [16] and polyamidoamine (PAMAM)). Although promising, the nonviral gene delivery systems have hitherto achieved moderate success due to their relatively low transfection efficiency and the safety issues associated with the carrier materials. For nonviral vectors, several hurdles are to be overcome at the cell level before efficient gene transfection is achieved. Ideally, nonviral vectors should protect the plasmid against degradation, lack cytotoxicity, facilitate cell entry of the plasmid, escape endo/lysosome entrapment, and enable the nuclear targeting of the plasmid [17]. Moreover, vectors are expected to sustain plasmid release for maintaining stable gene expression over prolonged periods of time. Sustained gene expression is particularly important for posterior ocular disorders where frequent intravitreal injections can cause serious side effects including retinal detachment [18].

**Figure 1:**
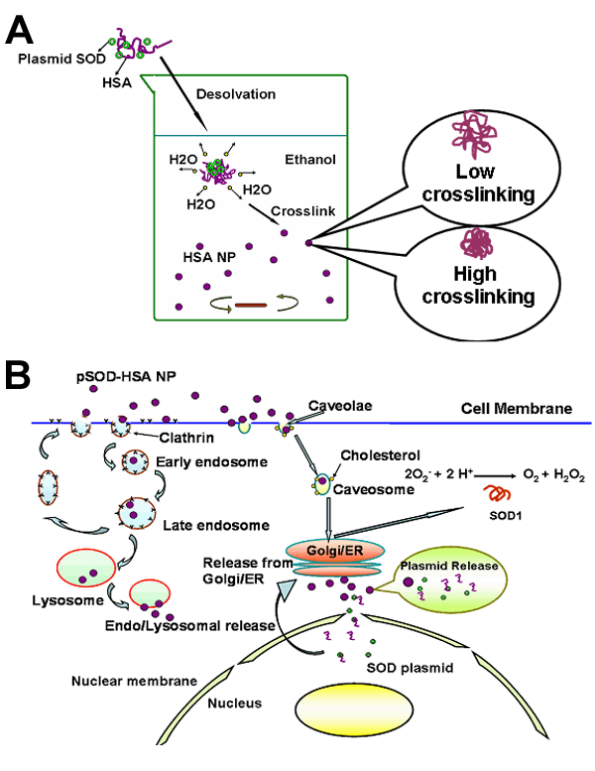
Albumin nanoparticles for retinal gene delivery. **A**: Scheme for the formation of pSOD-human serum albumin nanoparticles (HSA NP) using desolvation-crosslinking method. **B**: Proposed mechanisms for the endocytosis and activity of pSOD-HSA NP.

Among the aforementioned nonviral vectors, PLGA, the only material approved for human application, is criticized for incomplete DNA release at a rate far too slow compared to the cell turnover rate [19], restricting the full utilization of the plasmid dose. Therefore, new nonviral vectors are to be developed to overcome such problems and realize efficient gene delivery. Human serum albumin (HSA), although approved for parenteral use in humans, remains a less explored area for gene delivery. In this report, we use HSA as the sole carrier material in nanoparticles. HSA, composed of 585 amino acids with a molecular weight of 66 kDa (pI 5.8), has the unique ability to reversibly or covalently bind a variety of endogenous or exogenous ligands such as long chain fatty acids, vitamins, hormones, and metal ions with high affinity; this allows HSA to serve as a transport and depot protein for numerous compounds [20]. HSA binding often results in an increased solubility in plasma, decreased toxicity, protection against oxidation, and prolonged in vivo half-life of the bound ligands [21]. The cellular uptake of HSA via receptor (glycoprotein 60, also known as albondin)-mediated endocytosis [22,23] and the endogenous nature of this protein might render it highly efficient and safe as a carrier for gene delivery. In the eye, serum albumin filtrates through the blood-aqueous barrier and is the major component (50% of total proteins) of aqueous humor, which bathes the anterior side of the lens and provides important metabolites for the growth of rapidly dividing lens epithelial cells [24]. Significant amount of serum albumin was shown to pass through the aqueous humor into lens cells and transport metabolites between lens cells, possibly via a transcytotic route [25], indicating the presence of specialized uptake mechanisms in the cells of the eye and justifying the application of HSA for ocular delivery of therapeutic agents. Arnedo et al. reported that HSA nanoparticles improved the stability and nuclear accumulation of an oligonucleotide in lung fibroblast (MCR-5) cells [26]. The research of Carrabino et al. revealed that albumin boosts gene transfection efficiency of a plasmid DNA-cationic polymer complex in respiratory epithelial cells [27]. Our study is the first to use HSA as single carrier material for gene delivery. In this study, we prepared HSA nanoparticles containing a plasmid encoding the genes for SOD1 and enhanced yellow fluorescence protein (pSOD) and demonstrated that these particles enter cells via caveolae or clathrin mediated pathways, protect plasmid from biodegradation, allow plasmid escape from lysosome entrapment, sustain plasmid release, allow nuclear entry of HSA and potentially plasmid, and enhance gene expression and activity (Figure 1).

## Methods

### Materials

Human serum albumin, FITC-labeled human serum albumin (FITC-HSA), absolute ethanol, 25% (w/v) glutaraldehyde, trypan blue, DNase I, propidium iodide, filipin, chlorpromazine hydrochloride, paraformaldehyde, Triton X-100, thiazolyl blue tetrazolium bromide (MTT) salt, kanamycin, Hoechst 33258, protease, HEPES, potassium acetate, magnesium acetate, dithiothreitol (DTT), cytochalasin B, phebylmethanesulfonyl fluoride (PMSF), aprotinin, leupeptin, pepstatin, sodium acetate, ethylene glycol-bis(2-aminoethylether)-N,N,N',N'-tetraacetic acid (EGTA), adenosine 5'-triphosphate (ATP), creatine phosphate, creatine phosphokinase, and protease (P8083, bacterial, 8.1 units/mg solid) were products of Sigma-Aldrich Co. (St. Louis, MO). Lipofectamine^TM^ transfection reagent, DMEM/F12 cell culture medium and LysoTracker Red are products of Invitrogen Co. (Carlsbad, CA). MicroBCA protein assay kit was provided by Alkermes Inc. (Cambridge, MA). ARPE-19 cells were purchased from American Type Culture Collection (ATCC, Manassas, VA). A Cu, Zn superoxide dismutase and yellow fluorescent protein fusion plasmid (pSOD-EYFP) was engineered for use in these studies. Sterile deionized water was used for all investigations.

### Preparation of pSOD-EYFP plasmid

SOD1 cDNA was amplified by PCR and subcloned into pEYFP-N1 (Clontech, Palo Alto, CA, USA) at the Bam HI and NheI sites. This vector contains the CMV (immediate early promoter) upstream of the MCS. *Escherichia coli* transformed with the SOD1-EYFP-N1 plasmid (pSOD) was amplified by overnight culturing in LB medium supplemented with 1 mg/ml of kanamycin. The plasmid was collected and purified using Endofree Qiagen Plasmid Giga kit (Qiagen Inc., Valencia, CA) according to the manufacturer's protocol. Purity of the resulting plasmid was determined by the OD_260_/OD_280_ ratio (about 1.8-2.0).

### Preparation of pSOD-encapsulated human serum albumin nanoparticles

pSOD-encapsulated HSA NP was prepared using a modified desolvation-crosslinking method [28]. Briefly, 1 ml of HSA aqueous solution (2%, w/v) was incubated with 350 μg of pSOD solution in Tris-EDTA (TE) buffer (pH 8.0) for 5 min at room temperature. The mixture was subsequently added dropwise into ethanol (2 ml) under magnetic stirring (1,000 rpm). HSA nanoparticles formed were immediately cross-linked by the addition of either 1 or 20 μl of 10% (v/v) glutaraldehyde. The NP suspension was stirred for 12 h. Two ml of ethanol was added to the NP suspension to remove excess glutaraldehyde. The nanoparticles were separated by centrifugation at 48,000x g, 4 °C for 50 min (Sorvall Evolution RC®, Thermo Electron Co., Asheville, NC). The resulting NP pellet was resuspended in deionized water by vortexing (Scientific Industries Inc., Bohemia, NY).

### Characterization of pSOD-human serum albumin nanoparticles

pSOD- HSA NP were diluted in deionized water at a volume ratio of 1: 50 and characterized for particle size and zeta-potential using a ZetaPlus analyzer (Brookhaven Instrument Corporation. Holtsville, NY). Transmission electron microscopic image of HSA NP was acquired with an electron microscope (Philips-410LS, FEI Co., Hillsboro, OR) after the nanoparticles were negatively stained with Vanadium (NanoVan®, Nanoprobes, Inc., Yaphank, NY).

For encapsulation efficiency determination, freshly prepared pSOD-HSA NP suspension was centrifuged at 48,000x g, 4 °C for 50 min. The unencapsulated pSOD in the supernatant was quantified by reacting with equal volume of Hoechst 33258 solution (2 μg/ml, in TNE buffer) for 5 min at room temperature using the supernatant of blank HSA NP as a control. Fluorescence intensity of the resulting mixture was measured at 350 nm (excitation) and 450 nm (emission). The encapsulation efficiency of three batches of pSOD in HSA NP was calculated by the following equation:

Encapsulation efficiency(%)=(Plasmid used-unencapsulated plasmid)x100/Plasmid used

### In vitro release of pSOD-human serum albumin nanoparticles

For in vitro release study, the NP pellet (containing around 300 μg pSOD) was redispersed in 1 ml of phosphate buffer saline (PBS, pH 7.4) containing 0.1% (w/v) sodium azide and shaken at 100 rpm, in a shaker maintained at 37 °C. At predetermined time intervals, the particle suspension was spun down at 48,000x g, 4 °C for 50 min. pSOD released in the supernatant was quantified using Hoechst assay. The pellet was redispersed in 1 ml of fresh PBS for continuation of the release study. Cumulative release of pSOD as a percentage of encapsulated plasmid was calculated as a function of time.

### Nuclease and vitreous liquid stability of pSOD in human serum albumin nanoparticles

HSA NP (containing 1 μg of pSOD) was incubated at 37 °C for 0.5 h with either 5 μl of DNase I solution (0.1 unit/ml) or 5 μl of protease (10 mg/ml in PBS, pH 7.4), or 5 μl of fresh, homogenized vitreous humor obtained from bovine eyes. The DNase I degradation was terminated by adding 5 μl of 20 mM EGTA solution (pH 8) as DNase I inhibitor, followed by nanoparticle digestion with 5 μl of protease (10 mg/ml, essentially DNase-free) for 1 h at 37 °C. The integrity of the plasmid DNA was subsequently analyzed by gel electrophoresis in a 1% agarose gel at 80 V for 1.5 h.

### Cytotoxicity of human serum albumin nanoparticles

Safety of HSA NP as a gene delivery vector was evaluated by determining its cytotoxicity in ARPE-19 cells using MTT assay. Lyophilized blank HSA NP (prepared following the same protocols as pSOD-HSA NP, without the addition of pSOD) were dispersed to concentrations ranging from 5 to 5000 μg/ml in DMEM/F12 medium containing 10% FBS and supplemented with 100 mg/l of penicillin and 100 mg/l of streptomycin. ARPE-19 cells were seeded in a 96 well-plate at a density of 10,000 cells/well. Twenty-four hours post-seeding, the cells were washed thrice with PBS and incubated with HSA NP suspensions for up to 96 h. At the end of incubation, cells were treated with MTT salt (5 mg/ml in PBS) at 37 °C for 3 h. The relative cell viability was measured by dissolving the formazan crystals produced in 150 μl of DMSO and measuring absorbance at 550 nm, with untreated cells considered as 100% viable.

### Mechanism of cellular uptake of human serum albumin nanoparticles

FITC-labeled pSOD-HSA NP formulation was prepared following the procedures described in 2.3 except that 5 mg of FITC-labeled HSA was added into the HSA solution before NP preparation.

ARPE-19 cells were seeded in 96-well plate at a density of 1x10^5^ cells/well. On the third day post-seeding, the cells were washed thrice with PBS followed by equilibration in 200 μl of serum-free medium at 37 °C or 4 °C for 30 min. Uptake at 37 °C was initiated by replacing the serum-free medium with 100 μl of FITC-labeled pSOD-HSA NP suspension (0.1 mg/ml in serum-free medium) together with 100 μl of either serum-free medium, or filipin (1 μg/ml, an inhibitor for caveolae-mediated endocytosis), or chlorpromazine (50 μM, an inhibitor for clathrin-mediated endocytosis). Uptake at 4 °C was initiated by replacing the serum-free medium with 100 μl of FITC-labeled pSOD-HSA NP suspension (0.1 mg/ml) together with 100 μl of HSA solution (20, 10, and 5 mg/ml). Cells were incubated at 37 °C or 4 °C for 1 h. After removal of the NP suspension, cells were washed thrice with cold PBS followed by digestion in cell lysis buffer for 30 min. The fluorescence intensity was measured in a plate reader at 485 nm (excitation) and 535 nm (emission).

Uptake inhibition induced by chlorpromazine (50 μM) or filipin (1 μg/ml) was visualized by confocal microscopy. ARPE-19 cells were cultured on cover slips placed in 12-well plates at a density of 10,000 cells/well. Uptake inhibition was performed 3 days post-seeding, following the procedures described above. At the end of uptake, cells were washed thrice with cold PBS, followed by fixation with paraformaldehyde (4%) and permeabilization with 0.1% Triton X-100. The confocal images were acquired with a Carl Zeiss confocal microscope at 100X, following nuclei staining with propidium iodide (1 μg/ml).

### Endolysosomal escape of pSOD-human serum albumin nanoparticles

ARPE-19 cells were seeded on cover slip placed in a 12-well plate at 10,000 cells/well in 0.5 ml of culture medium. On the third day post seeding, cells were washed thrice with PBS followed by incubation with FITC-labeled pSOD-HSA NP (0.2 mg/ml) suspension containing 50 nM of LysoTracker Red for 1 h. The cells were then washed thrice with cold PBS and fixed with 4% of paraformaldehyde. Confocal microscopy was performed at 100X to locate the green fluorescent particles and red staining for late endosome-lysosome in the cytoplasm.

### Nuclear uptake of human serum albumin after digitonin permeabilization

Preparation of cytosol fractions: ARPE-19 cells growing to 85% of confluency in T-75 flasks were collected by trypsinization and centrifugation (1,000x g, 4 °C, 5 min). Cell pellet was washed twice with a washing buffer (10 mM HEPES, pH 7.3, 110 mM potassium acetate, 2 mM magnesium acetate, and 2 mM DTT) and suspended in 2 ml of transport buffer (20 mM HEPES, pH 7.3, 110 mM potassium acetate, 5 mM sodium acetate, 2 mM magnesium acetate, 1 mM EGTA, 2 mM DTT and 1 μg/ml each of aprotinin, leupeptin, and pepstatin). Cells were lysed by 5 strokes with a homogenizer (Tissue Tearor®, Biospec Products Inc., Bartlesville, OK) at an output of 20 Watts. The resulting homogenate was centrifuged at 1,500x g, 4 °C for 15 min to remove nuclei and cell debris. The supernatant was centrifuged at 45,000x g, 4 °C for 60 min. The final supernatant was dialysed against trnasport buffer followed by protein content evaluation using Micro BCA assay kit and used to maintain the stable composition of cytoplasm during nuclear uptake study post permeabilization.

Cell permeabilization and nuclear uptake of FITC-HSA: ARPE-19 cells grown on cover slip were rinsed in cold transport buffer followed by immersion in ice-cold transport buffer containing 40 μg/ml digitonin diluted from a 20 mg/ml stock solution. The cells were allowed to permeabilize for 5 min, after which the digitonin containing buffer was replaced with cold transport buffer. The cover slip was then blotted and incubated at 37 °C for 30 or 60 min with complete transport buffer containing 0.5 mg/ml of FITC-HSA. The complete transport buffer is composed of 50% cytosol, 20 mM HEPES, pH 7.3, 110 mM potassium acetate, 5 mM sodium acetate, 2 mM magnesium acetate, 1 mM EGTA, 2 mM DTT, 1 mM ATP, 5 mM creatine phosphate, 20 U/ml of creatine phosphokinase and 1 μg/ml each of aprotinin, leupeptin, and pepstatin.

At the end of incubation, the cells were washed thrice with 1 ml of cold transport buffer and fixed with 4% paraformaldehyde at room temperature for 10 min. Cells were then washed thrice with transport buffer and visualized using confocal microscope.

### In vitro transfection

ARPE-19 cells were seeded onto a 24-well plate at a density of 5x10^4^ cells/well 24 h prior to the transfection experiment. The transfection was initiated by washing the cells twice with 1 ml of sterile PBS followed by incubation at 37 °C with (1) 400 μl of serum-free culture medium; (2) 5 μg of free pSOD in 400 μl serum-free medium; (3) 1 μg of pSOD with 7.5 μl of Lipofectamine in 400 μl of serum-free medium (4) HSA NP containing 1 μg of plasmid DNA in 400 μl of serum-free medium, (5) blank HSA NP in 400 μl of serum-free medium at the equal HSA NP concentration as in (4). Treatments were terminated after 6 h incubation and the cells were cultured in complete medium for another 42 h. Cells were trypsinized and collected by spinning down at 1,500x g, 4 °C for 5 min. The cell pellet was redispersed in 0.5 ml of PBS and analyzed with a flow cytometer (Becton Dickinson FACScalibur, BD Biosciences Inc., Rockville, MD) for EYFP expression at 10,000 cell count/sample.

### Superoxide dismutase 1 activity evaluation

ARPE-19 cells were seeded on 6-well plate at a density of 2x10^5^ cell/well. Twenty-four hours post seeding, cells were transfected for 6 h with pSOD, pSOD-encapsulated in HSA NP or pSOD with 37.5 μl Lipofectamine, at a plasmid dose of 5 μg, in serum-free medium. Serum-free medium and blank HSA NP (at concentration equivalent to pSOD-HSA NP) were incubated with the cells serving as control. At the end of transfection, treatments were replaced by complete medium and incubated for another 42 h.

For SOD1 activity evaluation, cells were lysed and collected with a rubber policeman followed by centrifugation at 1,500x g, 4 °C for 10 min. The supernatant was subsequently centrifuged at 100,000x g, at 4 °C for 10 min. The supernatant was evaluated for SOD1 activity evaluation using a SOD activity assay kit (Cayman Chemicals Inc., Ann Arbor, Michigan) according to the manufacturer's protocol.

### Intravitreal administration of pSOD-loaded human serum albumin nanoparticles

Eighteen mice approximately 6 months old were injected IP with anesthesia, then they were divided into three groups: PBS, pSOD, and pSOD-HSA NP. Five μl PBS or about 5 μl pSOD (about 130 ng) or NP-plasmid (about 130 ng) was injected intravitreally, at approximately 2-3 mm posterior to the limbus in the left eye. Half of the mice in each group were sacrificed on day 2 (48 h) and half of the mice were sacrificed on day 7. After euthanasia using CO_2_, the left eye was immediately removed, washed in PBS, and the retina was isolated. The tissues were stored at -70 °C in lysis buffer containing 50 mM Tris/Cl pH 7.4, 1% Triton X-100, 0.5 mM EDTA and EGTA, 1:500 Sigma protease inhibitor cocktail, and 1:1000 phosphatase inhibitor.

The retinas were homogenized in about 70 μl lysis buffer with a sterile needle repeatedly and subjected to multiple freeze/thaw cycles. After the tissue was thoroughly homogenized, the mixture was centrifuged for 1 min at 500x g to settle insoluble debris. The supernatant was used for western blot analysis with Mouse anti-GFP antibody (BD Biosciences, San Jose, CA) and rabbit anti-SOD antibody (Abcam Inc., Cambridge, MA). In western blot analysis, GFP-SOD transfected N/N1003A lens epithelial cells were used as positive controls. For loading controls, 15 μg of β-actin was reacted with polyclonal β-actin antibody (Abcam, Inc.).

### Statistical analysis

Data are presented as mean±standard deviation, and analyzed by one way ANOVA with the Tukey's test applied post hoc for paired comparisons of means (SPSS 11, SPSS Inc., Chicago, IL).

## Results & Discussion

### Preparation of SOD1-plasmid containing human serum albumin nanoparticles

In this study, a desolvation-crosslinking process was employed for the preparation of HSA nanoparticles (HSA NP) [26,29] as illustrated in Figure 1A, with major modification in two aspects: first, 2% (w/v) HSA aqueous solution was added to two volumes of ethanol under high speed magnetic stirring. The previously reported method, on the other hand, added ethanol to aqueous HSA solution [29]. In our procedure, with rapid escape of H_2_O to the organic phase, the surface tension of the protein molecules promotes the instant formation of nanoparticles. Second, 0.1 instead of the reported 0.002 mg glutaraldehyde/mg HSA [29], the NP crosslinking reagent, was used. This procedure reduces the HSA NP size from the reported >200 nm to about 120 nm, as shown in Table 1. Further, the pSOD encapsulation efficiency increased from 64 to 84%, with an increase in the extent of crosslinking. As shown in Table 1, the size of pSOD-HSA NP decreased from 160 to 120 nm when the amount of glutaraldehyde used was increased from 0.002 to 0.1 mg/mg protein, which was accompanied by a decrease of zeta potential from -20 to -44 mV. The results indicate that high concentration of crosslinking reagent favors the formation of more condensed particles. Reduced particle size resulted in significantly increased surface area of NP, which allowed more surface adsorption of negatively charged pSOD and contributed to decreased zeta potential that is beneficial to the particle stability. Influence of pSOD encapsulation towards zeta potential was also reflected by the higher zeta potential of pSOD-loaded NP relative to blank NP.

**Table 1:** Characteristics of blank and pSOD-EYFP-encapsulated HSA NP formulations.

**Formulation**	**Size (nm)**	**Polydispersity**	**Zeta potential (mV)**	**pSOD loading%**
Blank HSA NP I*	158±3	0.16±0.01	-20.4±0.9	-
pSOD-HSA NP I*	157±1	0.09±0.01	-23.1±1.5	64.8±2.4
Blank HSA NP II**	116±7	0.29±0.04	-22.7±4.9	-
pSOD-HSA NP II**	122±2	0.25±0.01	-44.3±0.7	83.6±4.7

* Particles were prepared with 1 μl of 10% glutaraldehyde (low crosslinking). ** Particles were prepared with 20 μl of 10% glutaraldehyde (high crosslinking).

**Figure 2:**
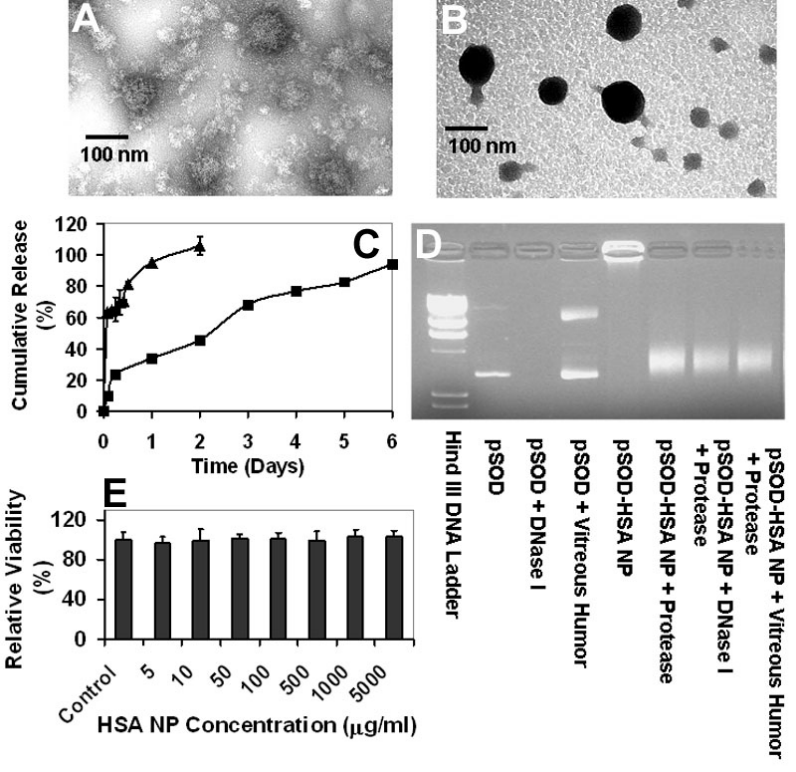
Albumin nanoparticles are non-toxic, protect plasmid from degradation, and sustain gene delivery. **A**: Human serum albumin nanoparticles (HSA NP) crosslinked with 1 μl of 10% (w/v) glutaraldehyde. **B**: HSA NP crosslinked with 20 μl of 10% (w/v) glutaraldehyde. Particles were negatively stained with Vanadium before transmission electron microscopic (TEM) imaging. **C**: HSA NP sustains in vitro plasmid release. Cumulative in vitro release (%) of pSOD from HSA NP with low and high crosslinking degree is shown. Data are presented as mean±SD, n=3. **D**: Enhanced stability of pSOD against DNase I and vitreous humor degradation after HSA NP encapsulation. Electrophoretic mobility analysis of pSOD-encapsulated HSA NP following 30 min incubation with nuclease, protease, or bovine vitreous humor. **E**: Lack of cytotoxicity of HSA NP in ARPE-19 cells. Relative viability of ARPE-19 cells treated with HSA NP for up to 96 h is shown. Cell viability was determined by MTT assay after HSA NP exposure. Data are expressed as mean±SD, n=8.

The structural difference of particles with low and high crosslinking degree was clearly evident in the TEM images (Figure 2A,B), where high crosslinking particles exhibited a more condensed structure compared with particles with low crosslinking degree.

### Human serum albumin nanoparticles sustain plasmid release

In vitro release of pSOD from HSA NP was evaluated by dispersing freshly prepared NP in 1 ml of PBS containing 0.1% sodium azide followed by shaking at 37 °C, 100 rpm. At predetermined time intervals, particle suspension was centrifuged at 48,000x g, 4 °C for 50 min, and the supernatant evaluated for plasmid concentration using Hoechst 33258. As shown in Figure 2C in vitro pSOD release from HSA NP with low crosslinking and a particle size of about 160 nm, is a biphasic curve including a fast-release phase, during which 65% of the drug load was released in the first 6 h, and a slow but steady release phase, accounting for the release of the remaining plasmid load in the next 44 h. The fast-release phase is attributable to immediate desorption of surface-adhered plasmid, and the dissociation of the loosely structured region in the nanoparticles. And the slow-release phase could be due to the release of tightly entrapped plasmid, accompanied with matrix expansion and/or degradation. On the other hand, highly crosslinked pSOD-HSA NP with a size of about 120 nm, showed continuous release profile, with substantially lower initial burst. In 6 h, 23% of loaded plasmid was released which was 60% less than the burst release of 160 nm NP. The rest of the DNA was released over a period of 6 days. On day 6, the cumulative release accounted for almost 100% of the plasmid encapsulated. Thus, high crosslinking degree enhances plasmid entrapment, surface negativity, and sustained gene delivery. Further, the particles allow complete release of the plasmid. Subsequent studies were primarily conducted with high crosslinking HSA NP, unless otherwise stated.

### Human serum albumin nanoparticles do not cause cytotoxicity

One critical requirement for nonviral gene vector is the ability to protect DNA against enzymatic degradation in the biological environment. The stability of HSA NP-encapsulated plasmid was evaluated by gel electrophoresis. pSOD-HSA NP was treated first with DNase I (0.1 unit/ml) or vitreous humor (from bovine eye) at 37 °C for 30 min. DNase I was then inactivated by 1 M of EGTA, and the NPs were degraded by incubation with 10 mg/ml protease solution at 37 °C for 1 h. The integrity of the released plasmid was determined by 1% agarose gel electrophoresis. As shown in the gel image (Figure 2D), similar DNA bands were observed for naked DNA and the lanes containing pSOD-HSA NP treated with protease alone, suggesting that the plasmid did not suffer major damage during NP preparation. Lane loaded with DNase I digested pSOD showed no band, indicating the complete degradation of the DNA, while plasmid treated with vitreous humor showed a major degradation band, indicating the instability of the DNA in the vitreous humor. After DNase I or vitreous humor and protease digestion, major DNA bands at a position similar to pSOD were observed, revealing that the encapsulated plasmid was intact after DNase I and vitreous humor digestion. The gel electrophoresis result indicates that HSA NP encapsulation enhances the plasmid stability and makes it more resistant to DNase I and vitreous humor degradation.

### Human serum albumin nanoparticles does not cause cytotoxicity

Cytotoxicity of blank nanoparticles was evaluated by incubating ARPE 19 cells at a series of concentrations for up to 96 h. The viability of HSA NP-treated cells was examined using MTT assay. As shown in Figure 2E, the percentage of viable cell was more than 90% over 96 h at NP concentrations up to 5 mg/ml. The highest concentration used in the cytotoxicity study is more than 100 fold higher compared to the particle concentration used for gene transfection, indicating that HSA NP is very safe as a vector for gene delivery.

### Human serum albumin nanoparticles enter cells via caveolae and clathrin mediated pathways

Intracellular trafficking route is a major concern for nonviral gene vectors. Pathways feasible for the cellular uptake of nonviral gene delivery vectors include: clathrin-mediated endocytosis, caveolae-mediated endocytosis, and clathrin-, caveolae-independent pathways [30]. It has been shown that many polycationic and targeting moiety-conjugated gene vectors are internalized by cells via a clathrin-mediated route [31,32]. In this route, gene vectors are rapidly transported from clathrin-coated vesicles to early endosomes (pH 6.3-6.8), and then to late endosomes within minutes [25]. After 10-25 min, the vectors are transferred to lysosome, where major plasmid breakdown occurs, contributing to low transfection efficiencies. Therefore, vectors capable of escaping clathrin-mediated endo/lysosomal entrapment and/or degradation will be advantageous for gene delivery. Caveolae, on the other hand are cholesterol-rich regions on the cell membrane. Cargos transported via caveolae-dependent route are delivered to caveosomes instead of lysosomes, and then to Golgi and endoplasmic reticulum (ER). Along this route, the pH is maintained neutral and no degradative end-station is reached [33], which is, therefore, more preferable for gene delivery.

**Figure 3:**
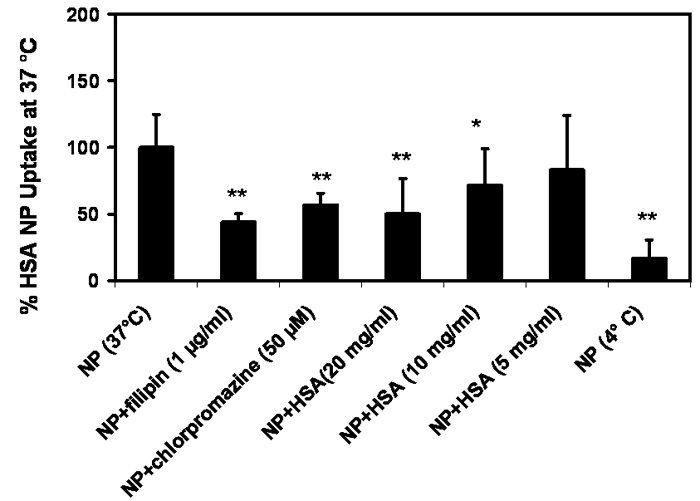
Cellular uptake of albumin nanoparticle is inhibited by low temperature, excess free albumin, filipin, and chlorpromazine. Cellular uptake of h uman serum albumin nanoparticles (HSA NP; 0.1 mg/ml) in 1 h at 37 °C in the absence or presence of filipin (1 μg/ml), chlorpromazine (50 μM), or free HSA (20, 10 and 5 mg/ml), and at 4 °C (Data are presented as mean±SD), n=9. * and ** indicate significant difference from the control group at p less than or equal to 0.05, and p less than or equal to 0.01, respectively.

**Figure 4:**
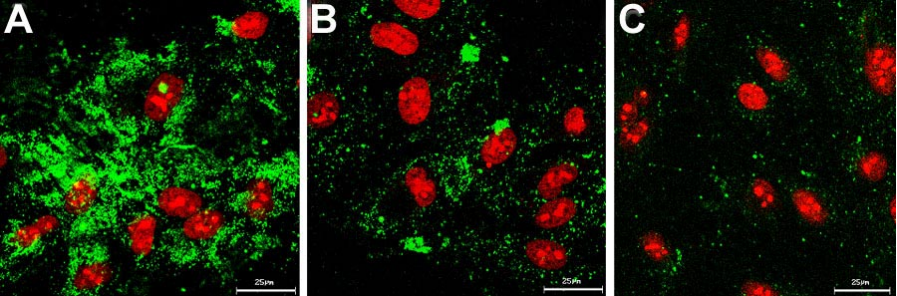
Albumin nanoparticles enter cells via caveloae- and clathrin- mediated endocytosis. Confocal image of cellular uptake of HSA NP (0.1 mg/ml) at 37 °C for 1 h **A** without uptake inhibitor; or in the presence of **B** chlorpromazine (50 μM), or **C** filipin (1 μg/ml).

We investigated the intracellular trafficking route of HSA NP by co-incubating FITC-labeled HSA NP with either filipin, the caveolae transport inhibitor, or chlorpromazine, the clathrin transport inhibitor. In addition, free HSA was added as a competitive substrate for membrane albumin-receptor at 37 °C. Further, influence of cellular energy deprivation on the particle uptake was assessed by using low temperature (4 °C). As shown in Figure 3, free HSA showed a concentration-dependent inhibition to the cell-associated fluorescence. At 20 mg/ml, free HSA reduced the cell-associated FITC-HSA NP by 50%. Reduction in the uptake temperature from 37 to 4 °C led to 84% decrease in particle uptake, revealing that the endocytosis of HSA NP is a receptor-mediated, energy-dependent event. At 37 °C, addition of either filipin (1 μg/ml) or chlorpromazine (50 μM) concomitantly with HSA NP produced 56 and 44% inhibition in the particle uptake, respectively. The results indicate that both caveolae and clathrin might be involved in the endocytosis of HSA NP, with caveolae potentially playing a more important role. This conclusion is further confirmed by confocal microscopy. As shown in Figure 4, the cell-associated green fluorescent NP was reduced by chlorpromazine and filipin, with filipin exerting a more evident inhibition. It was reported that filipin substantially reduces the transcelluar transport of albumin in cultured cells and the permeability of albumin through microvessels in rat lung, revealing that the caveolae-dependent route plays a major role in the cellular uptake of HSA [34]. Blomhoff et al. reported that formaldehyde-modified albumin can be internalized by scavenger receptors and degraded in lysosome, suggesting the involvement of clathrin route [35]. The authors argued that formaldehyde modification led to increased negative charge to albumin molecule, which directs the molecules to scavenger receptors and subsequently to the lysosomes, the common cell surface binding pathway that mediates the uptake and degradation. Our studies show the involvement of both caveolae and clathrin pathways for the uptake of HSA NP.

### Human serum albumin nanoparticles escapes endosome/lysosome entrapment and HSA enters cell nucleus

**Figure 5:**
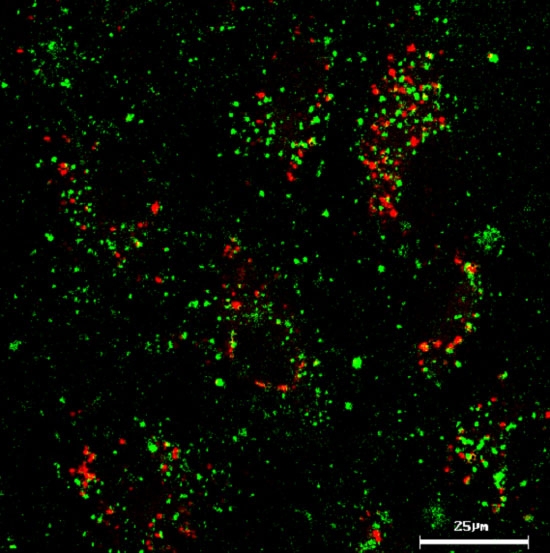
Albumin nanoparticles do not co-localize with lysosomes. Lack of lysosomal co-localization of FITC-labeled pSOD-HSA NP (0.2 mg/ml) after 1 h incubation with ARPE-19 cells in the presence of 50 nM LysoTracker Red.

To explore the potential of HSA NP to escape lysosome entrapment, uptake of FITC-labeled pSOD-HSA NP was performed in the presence of LysoTracker Red, a red fluorescent marker stains specifically late-endosomes or lysosomes. Co-localization of the two fluorophores was analyzed by confocal microscopy. Results obtained revealed no co-localization of the green particle and red endo-lysosome after 1 h incubation, as shown in Figure 5, indicating that pSOD-HSA NP likely entered the cells via caveloae pathway. Alternatively, the particles may have entered the cell via clathrin-dependent endocytotic pathways and then subsequently late endosomes and lysosomes. From late endosomes and lysosomes, the HSA NP may have rapidly escaped. The mechanism for the escape of the particles from endo/lysosome or other intracellular vesicles is unknown at this stage.

**Figure 6:**
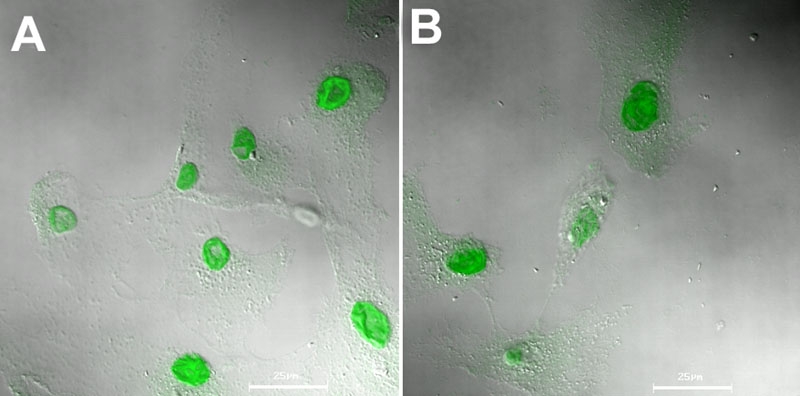
Albumin accumulates rapidly in the cell nucleus. Nuclear accumulation of FITC-labeled human serum albumin (0.5 mg/ml) in **A** 30 and **B** 60 min at 37 °C after digitonin (40 μg/ml, 0 °C, 5 min) permeabilization of ARPE-19 cells.

To study the potential of HSA for nuclear transport, cells were permeabilized with digitonin, a weak nonionic detergent that selectively perforates cell membrane while leaving the nuclear envelope and other subcellular organelles intact [36]. As shown in confocal images (Figure 6), 30 min incubation with FITC-HSA resulted in strong nuclei-associated green fluorescence in permeabilized ARPE-19 cells, indicating that free HSA molecules can be internalized by cell nuclei. Although the mechanism of the HSA-nuclear transport is unknown, it might contribute to the nuclear uptake of HSA NP and/or fragments of nanoparticles such as HSA-plasmid complexes. Possibly for this reason, investigators previously observed that HSA can enhance gene transfection capability of polyethyleneimine-DNA nanoparticles when admixed with the formulation [37]. Thus, the nuclear uptake study revealed the potential of HSA as a molecule capable of nuclear-transport and hence, nuclear drug/gene delivery.

### Human serum albumin nanoparticles facilitate gene expression in vitro and in vivo

**Figure 7:**
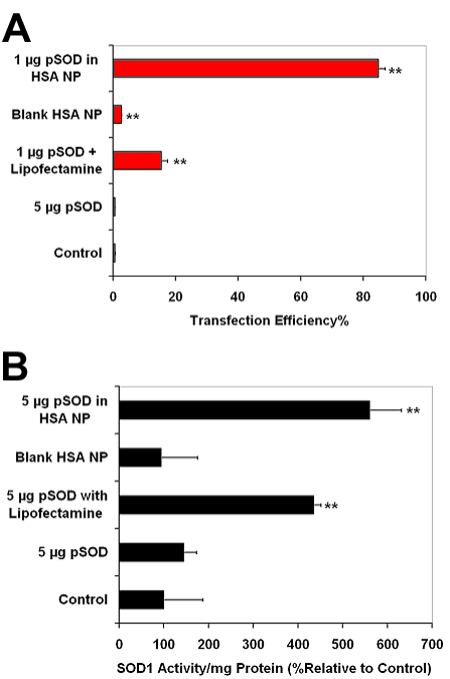
Albumin nanoparticles allow efficient in vitro gene transfection and expression. **A**: In vitro transfection efficiency with pSOD-encapsulated human serum albumin nanoparticles (HSA NP) in ARPE-19 cells. Following 6 h incubation with the gene delivery system, transfection was assessed at 48 h post transfection using flow cytometry (10,000 cell count per event). Data are expressed as mean±SD for n=3. ** Indicates significant difference from the control group at p less than or equal to 0.01. **B**: Enhanced SOD1 activity after cell transfection with 5 μg of pSOD encapsulated in HSA NP. Following 6 h incubation with the gene delivery system, SOD activity was assessed 48 h post transfection using a SOD activity assay kit. Data are expressed as mean±SD for n=3. ** Indicates significant difference from the control group at p less than or equal to 0.01.

We studied the in vitro transfection efficacy of the pSOD-loaded HSA NP by incubating human retinal pigment epithelial (ARPE-19) cells with serum-free medium containing pSOD alone, pSOD in Lipofectamine or pSOD-HSA NP for 6 h, followed by 42 h incubation in complete medium. Cells transfected with Lipofectamine served as the positive control, and cells incubated with culture medium and naked pSOD served as negative controls. Transfection efficiency was expressed as the percentage of EYFP-positive cells in a cell population. Figure 7A shows negligible transfection for naked plasmid at a pSOD dose of 5 μg, suggesting that transfection with plasmid alone is unsuccessful. On the other hand, Lipofectamine with 1 μg of pSOD allows 15% transfection efficiency. Interestingly, pSOD-HSA NP results in 86% transfection efficiency, indicating that the HSA NP formulation is about 6 fold more efficient than Lipofectamine. Cells treated with blank HSA NP were also analyzed to quantify any background contribution to the fluorescence measurements. The results indicated 2% transfection efficiency, possibly due to the autofluorescence of glutaraldehyde modified protein, which is negligible compared to the transfection efficiency measures obtained with the plasmid containing HSA NP.

To assess SOD1 functional gene expression, the activity of Cu, Zn SOD was measured 48 h after gene transfection in ARPE-19 cells and compared to that of untreated cells. SOD1 activity of blank HSA NP-treated cells was also evaluated as a control to study the effect of the vector alone on SOD1 expression. As shown in Figure 7B, 6 h incubation with 5 μg pSOD-encapsulated in HSA NP produced 5 fold higher SOD1 activity compared to untreated cells. At same plasmid dose, Lipofectamine mediated transfection resulted in 4 fold higher activity compared to untreated cells. However, incubation with the same dose of naked pSOD or blank NP resulted in negligible variation in SOD1 activity. It can be concluded from the results that, in vitro, HSA NP is more efficient in mediating the transfection of functional gene than Lipofectamine.

**Figure 8:**
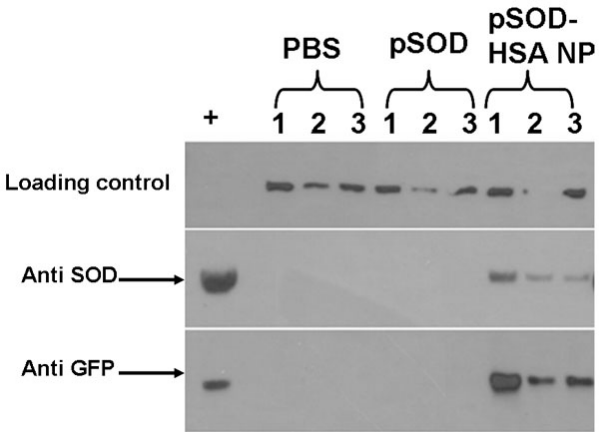
Albumin nanoparticles allow efficient in vivo retinal gene expression of SOD1. Western blot analysis of mouse retinas for SOD1 and EYFP on day 2 following intravitreal administration of PBS, or pSOD, or pSOD-HSA NP containing a 130 ng dose of pSOD. The + lane on the left indicates lysates from GFP-SOD transfected N/N1003A lens epithelial cells.

The in vivo transfection of the pSOD-loaded HSA NP was studied in mouse eye. This part of the study was performed at Kansas State University. Two days post intravitreal injection of 3 μl of HSA NP suspension at a dose of 130 ng pSOD per eye, the fusion protein was detectable using western blot in all eyes injected with the nanoparticle formulation (Figure 8). However, no fusion protein was detectable in animals treated with naked pSOD. It can, therefore, be concluded that the HSA NP formulation is effective for intravitreal gene delivery to the retina. Fusion protein was not detectable on day 7 in both plasmid treated and NP treated groups, indicating the need for further optimization of the non-viral vector for prolonged retinal gene expression. The HSA albumin vector might result in different durations of expression when injected in different locations. Following corneal intrastromal injection of a HSA nanoparticle formulation encapsulating a plasmid encoding for an intraceptor for vascular endothelial growth factor, we recently observed more prolonged expression, lasting for over a month [38]. Another possible explanation for the short duration of expression with intravitreal injection of HSA NP in the current study is that the formulation may have become less rigid in aqueous suspension form prior to in vivo injections, which occurred in about a week after the preparation of the nanoparticles. In future, we will compare freshly reconstituted lyophilized product with aqueous suspensions stored for different periods for their influence on in vivo gene expression.

The proposed mechanisms for the cell entry of pSOD-HSA NP, SOD1 gene transfection, protein expression, and effects are shown in Figure 1B.

### Conclusions

In summary, human serum albumin nanoparticle encapsulating plasmid encoding Cu, Zn SOD was developed using a modified desolvation-crosslinking method. Particles exhibited negligible cytotoxicity and could protect the plasmid DNA against the DNase I and vitreous humor degradation. HSA NP is also capable of sustaining plasmid release over 6 days in vitro. Cellular uptake of HSA NP is an energy-dependent process mediated by both caveolae- and clathrin-dependent endocytosis, and the HSA NP showed the potential to exist in a lysosome-free surrounding within the cell. Efficient gene transfection and gene expression was demonstrated under in vitro and in vivo conditions. Collectively, HSA NP overcomes several barriers associated with conventional nonviral gene delivery, and shows great potential as a gene delivery vector for successful gene therapy applications.
